# Manual hyperinflation in children

**DOI:** 10.5935/0103-507X.20210071

**Published:** 2021

**Authors:** Daiane Menezes Lorena, Maria Cecília Moraes Frade, Thalis Henrique da Silva

**Affiliations:** 1 Postgraduate Program, Faculdade de Medicina de Ribeirão Preto, Universidade de São Paulo - Ribeirão Preto (SP), Brazil.; 2 Postgraduate Program, Universidade Federal de São Carlos - São Carlos (SP), Brazil.

**Keywords:** Physical therapy modalities, Respiration, artificial, Respiratory therapy, Respiratory mechanics, Ventilators, mechanical, Intensive care units, pediatric, Child, Infant, newborn

## Abstract

Manual hyperinflation is used in neonatal and pediatric intensive care units to promote expiratory flow bias, but there is no consensus on the benefits of the technique. Thus, a review that presents supporting evidence is necessary. This study aims to review the literature on the manual hyperinflation maneuver in neonatal and pediatric intensive care units to analyze the evidence for this technique in terms of the forms of application (associated with other techniques or not), its safety, the performance of manual resuscitators and the influence of the physical therapist’s experience, in addition to evaluating the methodological quality of the identified articles. A search was performed in the following databases: Web of Science, ScienceDirect, PubMedⓇ, Scopus, CINAHL and SciELO. Two researchers independently selected the articles. Duplicate studies were assessed, evaluated by title and abstract and then read in full. The quality of the articles was analyzed using the PEDro scale. Six articles were included, two of which had high methodological quality. The main results provided information on the contribution of the positive end-expiratory pressure valve to increasing lung volumes and the use of chest compressions to optimize expiratory flow bias, the negative influence of operator experience on the increase in peak inspiratory flow, the performance of different manual resuscitators when used with the technique and the safety of application in terms of maintaining hemodynamic stability and increasing peripheral oxygen saturation. The available studies point to a positive effect of the manual hyperinflation maneuver in children who are admitted to intensive care units.

**Registration PROSPERO:** CRD42018108056.

## INTRODUCTION

In pediatric intensive care units (ICUs), many patients require invasive mechanical ventilation (IMV), a method that provides the child with adequate ventilatory support in conditions of respiratory failure due to pulmonary and nonpulmonary respiratory complications.^(^1^)^ Its goal is to maintain adequate gas exchange and decrease the work of the respiratory muscles and oxygen consumption.^(^2^)^

Although necessary, this support can cause complications due to the difficulty of eliminating bronchial secretions.^(^1^)^ This occurs due to changes in the natural mechanisms for the clearance of secretions in the airways, such as mucociliary transport and the cough reflex. The components that hinder these mechanisms include inadequate humidification, the use of sedatives and/or anesthetics, high inspired oxygen fractions, basal lung diseases and the presence of an artificial airway. In addition, the orotracheal tube (OTT) can cause microtraumas in the tracheal wall that favor the retention of pulmonary secretions.^(^3^-^5^)^

Ventilation-associated pneumonia (VAP) is an important and common complication in the pediatric population. It is the second most common infection associated with health care in pediatric ICUs; it is closely linked to the duration of IMV and has the effect of increasing the length of hospital stay.^(^6^)^ According to Willson et al., the incidence of pediatric VAP ranges from 2.9 to 45.1 per 1,000 days of ventilation, and VAP is related to morbidity and mortality in children.^(^7^)^

In this context, respiratory physical therapy aims to promote adequate bronchial hygiene in addition to reducing respiratory work, maintaining airway permeability and improving pulmonary ventilation and gas exchange.^(^8^,^9^)^ There are some airway clearance techniques that prevent, reduce and treat obstructions in this area, thereby reducing infections, the risk of mortality and the length of hospital stay. Several techniques used by physical therapists in pediatric ICUs have been documented in the literature, including manual hyperinflation (MH), which is routinely used in this environment.^(^10^)^

Manual hyperinflation aims to promote the removal of pulmonary secretions by increasing the peak expiratory flow (PEF). It consists of the use of a self-inflating manual resuscitator (MR), popularly known as an AMBU (artificial manual breathing unit), using slow inspiration with an inspiratory pause, followed by rapid expiration that may or may not be associated with chest compression. Its purpose is to expand the lung and increase the pulmonary distension pressure (transpulmonary pressure), which favors the increase of airflow to the atelectasis regions via the collateral channels and the redistribution and renewal of surfactant in the alveoli.^(^11^)^

Manual hyperinflation is widely used by physical therapists in several countries and is associated with airway clearance. In the study by Volpe et al.,^(^12^)^ the authors conclude that the technique promotes increased pulmonary compliance and oxygenation by generating an expiratory flow bias. This is described as the mean difference in PEF by peak inspiratory flow (PFI) of 33L/minute (PFE-PFI > 33L/minute); that is, the expiratory flow should exceed the inspiratory flow to promote cephalic movement of the mucus.^(^12^-^14^)^

Scientific evidence regarding the use of MH in the adult population is well established, but there are few studies in the pediatric population. Thus, this study aimed to conduct an integrative review of the available literature on the MH maneuver in pediatric and neonatal ICUs and to analyze the evidence regarding the MH maneuver in relation to its forms of application (with or without other techniques), its safety, the performance of self-inflating manual resuscitators and the influence of the experience of the physical therapist and to evaluate the methodological quality of the identified articles.

## METHODS

The review protocol was registered by the International Prospective Register of Systematic Reviews (PROSPERO), under number CRD42018108056.

The eligibility criteria were as follows: studies conducted in pediatric and neonatal ICUs in the last 8 years using the MH technique, original articles for which the full text was available, articles published in Portuguese or English, research characterized as clinical trials, longitudinal studies or cross-over studies.

The exclusion criteria were studies with samples that included adult ICU patients, review articles, duplicates, conference articles, editorials, comments or supplementary articles that did not address the proposed topic or were not available in full or in the predetermined languages.

The search was conducted in six databases: Web of Science, ScienceDirect, PubMedⓇ, Scopus, Cumulative Index of Nursing and Allied Health Literature (CINAHL) and Scientific Electronic Library Online (SciELO) and was conducted from June to September 2018. We used three descriptors: “Manual Hyperinflation” AND Pediatrics AND “Mechanical Ventilation” and included the operator “AND” to form the search string.

The analyzed outcomes were the forms of application of the MH maneuver (with or without other techniques), their safety, the performance of self-inflating manual resuscitators and the influence of the experience of the physical therapist.

First, the presence of duplicate studies was verified; then, the articles were evaluated by title and abstract and then by reading of the full text.

A computational tool called State of the Art through Systematic Review (StArt) was used to support the systematic review process. The tool can be used in three stages: during planning, through the completion of a protocol; during execution, when it can add and evaluate articles and extract information from those that are relevant; and during the data summary, through graphs and tables.^(^15^)^

The quality of the included articles was analyzed using the PEDro scale, which is used to evaluate the quality of controlled clinical trials. In systematic reviews, the PEDro scale total score is used to characterize the reliability of a study as “moderate” to “good”. It consists of 11 items, and the higher the score is, the better the article’s quality.^(^16^)^

Study selection (reading of the titles, abstracts and full texts) was performed by two independent researchers, and discrepancies between them were resolved by a third researcher. The same process was used to apply the PEDro scale to determine methodological quality.

## RESULTS

A total of 294 studies were found; of these, 100 were extracted from the Web of Science database, 59 from Scopus, 105 from ScienceDirect, 14 from PubMedⓇ and 16 from CINAHL. No articles were found in the SciELO database. Of the total number of studies, 10 were duplicates, and 284 remained potentially relevant.

**Figure 1:**
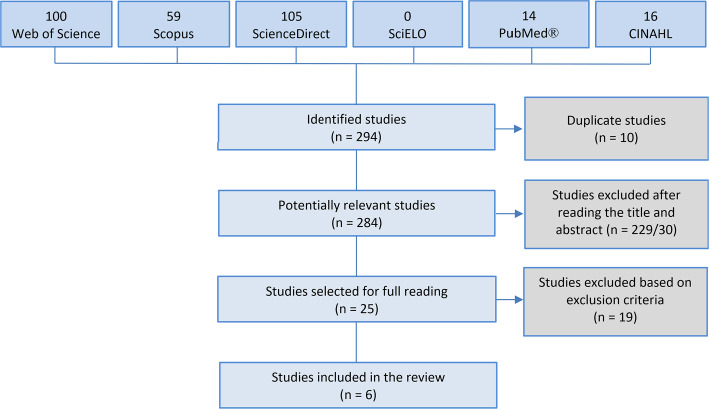
Study selection.

In the second stage, the titles and abstracts were read, and 229 and 30 articles were excluded, respectively. In the third stage, 25 articles were read in full, and 19 were excluded because they met exclusion criteria. Thus, six articles were included in this review (Figure 1).

Table 1 presents the characteristics of the included studies, including the study type, sample, sample size and intervention performed. Six clinical trials were included. Of these, one was of randomized, and two were cross-sectional and randomized.

The target population was children hospitalized in neonatal or pediatric ICUs and physical therapists who tested the MH maneuver in models of neonatal and pediatric lungs. The sample sizes ranged from 9 to 105 individuals. Only the study by Viana et al.^(^17^)^ included a sample calculation. Soudararajan et al.^(^18^)^ obtained a convenience sample, Novais de Oliveira et al.^(^19^)^ based their sample size on previous studies, and the other studies (Gregson et al., de Oliveira et al. and Koop et al.) did not address this issue.^(^5^,^20^,^21^)^

Briefly, the interventions performed involved the use of MH with or without the use of positive end expiratory pressure (PEEP) valve,^(^17^)^ MH with thoracic vibrocompression (TVC),^(^18^)^ and an analysis of the performance of three manual resuscitators from different manufacturers in terms of the ventilatory data of two test lungs (neonatal and pediatric) with different oxygen flow rates.^(^20^)^ In addition, the influence of professional experience on MH performance,^(^19^)^ the contribution of TVC to the increase in expiratory flow during MH^(^5^)^ and the clinical variables after MH was performed were analyzed.^(^21^)^

SciELO - Scientific Electronic Library Online; CINAHL - Cumulative Index of Nursing and Allied Health Literature.

**Table 1:** Characteristics of the included studies: type of study, sample, sample size and intervention performed

Author	Type of study	Sample	Sample size	Intervention
Gregson et al.^(^5^)^	Clinical trial	105 sedated children	Did not mention	MH was performed with or without TVC. The data were measured at three time points: (1) before the intervention, with the patient receiving mechanical ventilation; (2) while receiving MH and (3) while receiving MH associated with TVC during expiration
Viana et al.^(^17^)^	Randomized clinical trial	28 preterm neonates	28 individuals	In all patients, two respiratory physical therapy interventions were performed, and MH with and without the PEEP regulating valve was compared. The variables were measured 5 minutes before tracheal aspiration and at 1 and 30 minutes after aspiration
Soudararajan et al.^(^18^)^	Clinical trial	18 pediatric patients during the postoperative period of cardiac surgery	Convenience sample	MH followed by TVC was applied to all patients. Two physical therapists were needed; one performed MH, and the other performed TVC. The variables were measured before and 30 minutes after MH
Novais de Oliveira et al.^(^19^)^	Randomized crossover clinical trial	22 physical therapists	Based on previous studies	Two groups (experienced and inexperienced physical therapists) simulated MH in two neonatal test lungs (neonatal and pediatric) in two clinical situations each (one healthy lung and one with decreased compliance). The MRs were from 3 different manufacturers
de Oliveira et al.^(^20^)^	Cross-sectional randomized clinical trial	22 physical therapists	Did not mention	The performance of 3 MRs from different manufacturers was evaluated with two test lungs (neonatal and pediatric) and with different oxygen flow rates. Two situations were simulated: healthy (normal respiratory mechanics) and restrictive (decreased lung compliance). The lungs were connected to a 100% oxygen source with oxygen flow rates of 0, 5, 10 and 15 L/minute. All of the physical therapists performed 10 manual hyperinflations with each of the 3 resuscitators for both the neonatal and pediatric lungs
Koop et al.^(^21^)^	Clinical trial	9 preterm newborns	Did not mention	In all patients, interventional neonatal physical therapy procedures were performed that included pulmonary auscultation, TVC, vibration and thoracoabdominal support. Subsequently, MH was applied, and the OTT was aspirated. Data were collected before the maneuver and at 1, 5 and 10 minutes after the intervention

MH - manual hyperinflation; TVC - thoracic vibrocompression; PEEP - positive end-expiratory pressure; MR - manual resuscitator; OTT - orotracheal tube.

Regarding the method of application of the maneuver, four studies mentioned thus of slow insufflation with and inspiratory pause ranging from 2 to 3 seconds, followed by rapid release during the expiratory phase.^(^17^,^19^-^21^)^ Three studies reported the use of chest compressions during expiration.^(^17^,^18^,^21^)^ Only one study did not mention the method of application of MH.^(^5^)^

Regarding the analyzed variables, only the study by Koop et al. evaluated the patients’ vital data, such as heart rate (HR), respiratory rate (RR) and peripheral oxygen saturation (SpO2).^(^21^)^ Soudararajan et al. analyzed arterial oxygen pressure (PaO2) and chest radiographs 30 minutes after MH.^(^18^)^ Gregson et al., Viana et al., Novais de Oliveira et al. and de Oliveira et al. focused mainly on ventilatory data, such as inspiratory pulmonary volume (PVinsp), expiratory lung volume (PVexp), inspiratory pulmonary resistance (PRinsp), expiratory pulmonary resistance (PRexp), tidal volume (Vt), inspiratory pressure peak, PIF, PEF and inspiratory time (Tinsp).^(^5^,^17^,^19^,^20^)^ Only Gregson et al. evaluated the force applied by the operator during chest compressions in MH.^(^5^)^

Regarding the results, in general, there was a positive effect of MH on the analyzed variables. The studies showed increased lung volumes during MH with and without the use of a PEEP valve, increased PaO_2_ and improved chest radiography in children with pulmonary collapse after cardiac surgery.^(^17^,^18^)^ In addition, there was a difference in performance between neonatal and pediatric MRs and an increase in ventilatory parameters according to the increase in oxygen flow rate.^(^20^)^ There was also a higher PIF when MH was performed by experienced physical therapists and an increase in expiratory flow bias when MH was combined with TVC.^(^5^,^19^,^20^)^ Finally, there was an increase in HR and RR in the first minute after MH and an average increase in SpO2 of 0.76% at each evaluation time point.^(^21^)^

To analyze the methodological quality of each article included in this review, the PEDro scale was used, as described in table 2. Each item was flagged in the studies, and a final score was recorded. The maximum score of this scale is 11; however, for the included studies, the maximum score was nine, and the minimum was four.

**Table 2:** PEDro scale

PEDro scale	References					
	Viana et al.^(^17^)^	Soudararajan et al.^(^18^)^	de Oliveira et al.^(^20^)^	Novais de Oliveira et al.^(^19^)^	Gregson et al.^(^5^)^	Koop et al.^(^21^)^
1. The eligibility criteria were specified	✓	✓	X	X	✓	✓
2. The subjects were randomly allocated to groups	✓	X	X	X	X	X
3. The allocation of subjects was concealed	✓	X	✓	X	X	X
4. The groups were similar at baseline for the most important prognostic indicators	X	X	X	✓	X	X
5. There was blinding of all subjects	✓	X	X	X	X	X
6. There was blinding of all therapists who administered the therapy	X	X	✓	X	X	X
7. There was blinding of all assessors who measured at least one key outcome	✓	X	X	X	X	X
8. Measurements of at least one key result were obtained in more than 85% of the subjects initially allocated to the groups	✓	✓	✓	✓	✓	✓
9. All subjects for whom results were measured received the treatment or control condition according to the distribution or, when this was not the case, the data were analyzed for at least one of the results-key for an intention-to-treat analysis	✓	✓	✓	✓	✓	✓
10. The results of between-group statistical comparisons were reported for at least one key outcome	✓	X	✓	✓	✓	✓
11. The study provided both point measures and variable measures for at least one key outcome	✓	✓	✓	✓	✓	X
Total score	9/11	4/11	6/11	5/11	5/11	4/11

✓ - scored; X - not scored.

## DISCUSSION

Manual hyperinflation is a widely known technique used in neonatal and pediatric ICUs, specifically for children under IMV. Through a search of the aforementioned databases, we sought to gather information on the main parameters demonstrating the effect of the use of this technique in the aforementioned population.

There are still few studies involving MH in this context, and among those included in this review, it can be noted that MH was used to investigate different outcomes. While Viana et al. were interested in researching the use of the PEEP valve during MH, Soudararajan et al. considered blood gas analysis and chest radiography data.^(^17^,^18^)^ Novais de Oliveira et al. compared MH performed by experienced and inexperienced physical therapists, and de Oliveira et al. investigated the performance of neonatal and pediatric MRs.^(^19^,^20^)^ Gregson et al. analyzed the effect of chest compression during MH, and Koop et al. analyzed vital data after this maneuver.^(^5^,^21^)^

Regarding the form of application of MH, four studies mentioned performances similar to those described in the literature.^(^17^,^19^-^21^)^ Since the advent of MH, there has been interest in correctly detailing the technique to ensure that its goal of removing secretions is achieved. Experts recommend performing the maneuver as follows: first, slow insufflation of the resuscitator to a volume 50% greater than that provided by the ventilator, followed by a 2-second inspiratory pause and rapid release of pressure with or without chest compression to promote a high expiratory flow that shifts the secretions to the central airways and simulates the effect of coughing.^(^12^,^22^-^25^)^

Regarding the use of the PEEP valve, Viana et al. indicated that there was no significant difference in MH performed with and without the valve. However, they mentioned beneficial clinical evidence, such as increased lung volumes, when a PEEP valve was used.^(^17^)^ Another study, by de Santos et al., also used MH with a PEEP valve and obtained the same result of increased inspiratory and expiratory volumes, in addition to increased static compliance.^(^26^)^ Savian et al. found that the PEEP levels imposed during MH could alter the PEF and noted that, based on the type of circuit used with PEEP above 10 cmH_2_O, there is a decrease in PEF.^(^27^)^ Thus, when the patient does not require high PEEP values, positive effects of the using the PEEP valve during MH are observed that may reduce the deleterious effects of disconnecting the patient from the mechanical ventilator during the cyclical opening and closing of the lung units.^(^17^)^

Regarding the performance of MRs, the present review pointed to the differences in ventilatory parameters between the three brands of devices, both neonatal and pediatric; regarding oxygen flow, there was an increase in ventilatory parameters according to the increase in flow rate at oxygen flow rates of 0 and 15L/minute.^(^20^)^ In a further comparison of MR performance, the study by Jones et al. analyzed the differences between two MR circuits (Mapleson-CⓇ and MagillⓇ) in terms of the mobilization of pulmonary secretions through PEF measurements and inspiratory and expiratory relationships; among other results, they found that although the two circuits achieved ideal flows for secretion removal, the PEF produced by the Mapleson-CⓇ circuit was significantly higher, making it the most effective circuit for the investigated outcome.^(^28^)^ These results show the importance of tests and studies of MRs to identify the ones that are best qualified to achieve the purpose of the MH maneuver and yield benefits in clinical practice.

Regarding chest compression performed during MH, Gregson et al. showed that this factor contributes significantly to generating an expiratory flow bias that favors the displacement of pulmonary secretions to the central airways.^(^5^)^ In addition, Novais de Oliveira et al. reported that the use of MH alone does not seem to confer any therapeutic advantage in terms of mucociliary airway clearance but may contribute to recruitment.^(^19^)^ In the same vein, another study researched this issue and obtained the opposite finding: that MH with chest compression is hemodynamically safe but had no added benefit in terms of the optimization of oxygenation, respiratory mechanics and clearance of secretions from the bronchial tubes when performed by a single professional, with one hand providing MH and the other performing chest compressions.^(^4^)^ However, the same study noted that Vts below the recommended values were probably used, and the relationship between the expiratory and inspiratory flows generated during the execution of the maneuver was suboptimal. Finally, the authors emphasized that the lower the Vt is, the lower the thoracic expansion, the recruitment of collapsed units and the generated PEF are, making the technique ineffective.

Regarding the experience of the physical therapist, there was a significant increase in PIF when MH was performed by professionals with more experience with this maneuver. This result was not linked to the physical characteristics of the operator but to greater confidence of the operator, which causes him or her to be less careful when performing the maneuver and in turn reduces the benefits of the maneuver.^(^19^,^20^)^ The study by Volpe et al.,^(^12^)^ which included 12 physical therapists with a mean of 5 ± 3 years of experience in adult ICUs, stimulated the performance of MH the way they usually perform it in daily practice and after they received instruction based on recommendations; the study found that before they received the instruction, the professionals tended to perform the maneuver with high PIF. This can cause an insufficient expiratory flow bias and promote inspiratory flow bias; as a result, instead of reaching the central airways, the secretions can move deeper into the lungs if the patient is completely sedated and does not have a cough reflex. The study by Ortiz et al.,^(^14^)^ which included eight physical therapists with an average of 2.6 years of ICU practice, noted that most professionals performed MH with equally high PIFs. A possible explanation for this finding is that the guidance for physical therapists regarding MH does not emphasize the need for an expiratory flow bias, and, as a result, the professionals customized the maneuver in their clinical practice based on the impression that a high PIF (with two to three rapid compressions) stimulates coughing and increases mucociliary clearance. This information should draw physical therapists’ attention to the way they apply MH and should highlight the importance of training programs that teach professionals to perform MH according to expert recommendations to favor the removal of pulmonary secretions.^(^12^,^14^)^

Regarding the arterial blood gas data (such as PaO_2_), chest radiography and vital data (such as HR, RR and SpO_2_), it can be observed in the present study that MH had positive effects in the population of children admitted to the ICU. In studies conducted in various populations, it was observed that in patients with atelectasis, MH resulted in an improvement in radiographic signs, Vt and the relationship between PaO_2_ and the fraction of inspired oxygen (FiO2).^(^29^)^ Blattner et al. reported an increase in both PaO_2_ and static compliance, which reduced the weaning time from IMV.^(^30^)^ Regarding the association with hemodynamic data, which has been studied for several decades, the literature indicates no change in blood pressure, HR^(^31^-^34^)^ or increased arterial oxygenation;^(^24^)^ however, there may be a reduction in cardiac output after MH.^(^31^,^33^)^

In terms of methodological quality of the included articles, only one study had a high total score (nine out of 11 points).^(^18^)^ de Oliveira et al.^(^20^)^ scored six, while Novais de Oliveira et al.^(^19^)^ and Gregson et al.^(^5^)^ scored five points. Finally, Soudararajan et al. and Koop et al. scored four points, indicating low methodological quality.^(^18^,^21^)^ Thus, only two studies had high methodological quality (scores ≥ 6), and most (four of them) had low methodological quality (scores < 6), according to the categorization proposed by Moseley et al.^(^35^)^ In addition, all articles met Items 8 and 9 of the scale, which correspond to the measurement of at least one key result in more than 85% of the subjects initially distributed into the groups and the provision of the treatment or control condition to all subjects according to their allocation, respectively.

Regarding the limitations of the present review, the small number of studies on the topic of interest can be observed. Each of the included articles investigated different outcomes, which restricted the ability to compare the results. In addition, most of the articles had deficits in their methodological quality, which compromises their reproduction in clinical practice.

## CONCLUSION

Six studies on the topic of study were included; of these, only two had high methodological quality. The main results provided information on the contribution of the positive end-expiratory pressure valve to the increase in lung volumes and the use of chest compressions to optimize the expiratory flow bias, with increased peak expiratory flow; the negative influence of operator experience on the increase in peak inspiratory flow; the performance of different manual resuscitators during the performance of the technique and the safety of its application, with the maintenance of hemodynamic stability and increased peripheral oxygen saturation. Thus, currently available studies point to a positive effect of the manual hyperinflation maneuver performed in children admitted to intensive care units.
